# Supplementary material for: Learning-associated flexibility of cortical taste coding is impaired in Shank3 knockout mice
Source: eLife. 2026 Nov 12;14:RP108988. doi: 10.7554/eLife.108988 (PMC13759187; doi:10.7554/eLife.108988)
Supplement: Peer review history

# Editor report: eLife Assessment

**DOI:** 10.7554/eLife.108988.3.sa0

**Authors:** Brice Bathellier

**Affiliations:** Centre National de la Recherche Scientifique France

**Keywords:** Convincing, Valuable

This **valuable** study provides **convincing** evidence for deficits in aversive taste learning and taste coding in a mouse model of autism spectrum disorders. Specifically, the authors found that Shank3 knockout mice exhibit behavioral deficits in learning and extinction of conditioned taste aversion, and calcium imaging of the gustatory cortex identified impaired neuronal responses to taste stimuli. This paper will likely be of interest to researchers studying how learning and sensory processes are affected by genetic causes of autism spectrum disorders.

# Referee report: Reviewer #1 (Public review)

**DOI:** 10.7554/eLife.108988.3.sa1

Summary:

The study from Wu and Turrigiano investigates how disruption of taste coding in a mouse model of autism spectrum disorders (ASDs) affects aversive learning in the context of a conditioned taste aversion (CTA) paradigm. The experiments combine 2photon calcium imaging of neurons in the gustatory portion of the anterior insular cortex (i.e., gustatory cortex) with behavioral training and testing. The authors rely on Shank3 knockout mice as a model for ASDs. The authors found that Shank3 mice learn CTA more slowly and extinguish the memory more rapidly than control subjects. Calcium imaging identified impairments in taste evoked activity associated with memory encoding and extinction. During memory encoding, the authors found less suppressed neuronal activity and increased correlated variability in Shank3 mice compared to control. During extinction, they observed a faster loss of taste selectivity and degradation of taste discriminability in mutants compared to controls.

Strengths:

This is a well-written manuscript that presents interesting findings. The results on the learning and extinction deficits in Shank3 mice are of particular interest. Analyses of neural activity are well conducted and provide important information on the type of impaired cortical activity that may correlate with behavioral deficits.

Weaknesses:

The authors did an excellent job addressing the weaknesses highlighted in my first assessment.

# Referee report: Reviewer #2 (Public review)

**DOI:** 10.7554/eLife.108988.3.sa2

Summary

Wu and Turrigiano investigate how Shank3 loss affects experience-dependent changes in sensory representations during conditioned taste aversion learning and extinction. Using longitudinal two-photon calcium imaging in the gustatory cortex, the authors show that Shank3 knockout mice acquire taste aversion more slowly but, after additional conditioning, reach an aversion comparable to wild-type mice; this learned aversion then extinguishes more rapidly. At the neural level, knockout mice exhibit reduced stimulus-evoked suppression and increased correlated variability; while learning and extinction are accompanied by changes in the reliability, selectivity and population-level discriminability of taste representations. The revised manuscript more clearly distinguishes baseline genotype-dependent differences in cortical activity from learning-associated changes and appropriately frames the relationship between neural activity and behaviour as associative rather than causal.

Strengths

A major strength of the study is the combination of longitudinal cellular-resolution imaging with a behavioural paradigm that allows cortical population activity to be followed across acquisition, retrieval and extinction. This provides a rich description of how sensory representations evolve as learned value changes, and how these dynamics differ following Shank3 deletion. The observation that knockout mice eventually acquire a robust aversion but subsequently extinguish it more rapidly is particularly useful because it separates impaired acquisition from subsequent instability of the learned association.

The revised manuscript has substantially addressed several concerns raised in the original review. Importantly, the authors now show that reduced stimulus-evoked suppression is already evident during pre-learning habituation and that increased coactivity therefore appears to reflect, at least in part, a pre-existing network property rather than a consequence of learning. They additionally report that the level of coactivity at the beginning of conditioning correlates with subsequent behavioural acquisition, providing a useful link between individual variation in cortical activity and learning performance. This analysis strengthens the association between cortical network state and behaviour without establishing causality.

Another useful addition concerns the potential contribution of licking behaviour to taste decoding. Because sampling behaviour necessarily differs as animals acquire an aversion, separating sensory representations from movement-related activity is difficult in this paradigm. The authors now perform decoding during the ten-second post-sampling period and find above-chance decoding after licking has ceased, making it less likely that differences in licking alone explain the principal population-decoding results.

The manuscript is also clearer in its anatomical and conceptual terminology. The recordings are now appropriately described as being from gustatory cortex rather than implying coverage of the broader anterior insular cortex, and the conclusions have been restricted primarily to conditioned taste aversion rather than generalised to cognitive flexibility more broadly. The latter is particularly important because whether these findings generalise to reversal learning, probabilistic learning, or other forms of adaptive behaviour remains unknown.

Weaknesses

The principal remaining limitation is mechanistic. The experiments establish a robust association between Shank3 deletion, altered cortical activity and altered learning dynamics, but they do not establish the causal relationships among these observations. The new correlation between early coactivity and subsequent learning is informative, but manipulating the relevant network property would ultimately be required to determine whether increased correlated variability contributes directly to slower acquisition. The authors now acknowledge this limitation and have appropriately removed language implying causality.

Similarly, the cellular or circuit origin of the altered correlated variability remains unresolved. Reduced inhibition is an interesting potential explanation, but the authors do not directly measure interneuron function or inhibitory transmission here. Consequently, the proposed relationship between Shank3 loss, altered inhibition, increased correlated activity, and impaired sensory encoding should remain a hypothesis emerging from the results rather than a demonstrated mechanism.

A further limitation is the absence of a full conditioned-stimulus-only Shank3 knockout control group. The newly analysed habituation recordings partly address this issue by demonstrating reduced suppression and a tendency toward increased coactivity before learning, and the cross-session decoding analysis suggests that naïve knockout cortical populations can nevertheless distinguish water from saccharin. These analyses considerably improve interpretation of the existing experiment, although they are not equivalent to longitudinal comparison with a knockout control group undergoing the complete protocol without aversive conditioning.

Finally, the interpretation of population activity in terms of taste identity, learned value and their interaction remains necessarily limited by the task design. The results clearly demonstrate experience-dependent changes in population discriminability, but because taste identity, learned value, and sampling behaviour covary during conditioned taste aversion and extinction, the present experiments cannot fully separate the precise information represented by these population changes.

Overall assessment

The revision has addressed the major interpretational concerns raised in the previous review and has strengthened the manuscript through several useful additional analyses. In particular, distinguishing pre-existing cortical abnormalities from learning-associated changes, relating early coactivity to subsequent behaviour, controlling more carefully for licking-related activity, and removing causal language better align the conclusions with the evidence. The study therefore provides solid evidence for altered experience-dependent sensory coding and learning dynamics following Shank3 loss, while the mechanisms connecting these phenomena remain an important question for future work.

# Referee report: Reviewer #3 (Public review)

**DOI:** 10.7554/eLife.108988.3.sa3

In this study Wu & Turrigiano investigate an ethologically relevant form of associative learning (conditioned taste aversion-CTA) and its extinction in the Shank3 KO mouse model of ASD. They also examine the underlying circuits in anterior insular cortex (AIC) simultaneously, using two-photon calcium imaging through GRIN lens. They report that Shank3 KO mice learn CTA slower and suggest that this is mediated by a reduction in tastant-stimulus activity suppression of AIC neurons and reduced signal-to-noise ratio due to increased noise correlations in AIC neurons. Interestingly, once Shank3 KO mice do acquire CTA, they extinguish the aversive memory more rapidly than wild-type. This accelerated extinction is accompanied by a faster loss of neuronal and population-level taste selectivity and coding in the AIC compared to WT mice.

This is an important study that uses in vivo methods to assess circuit dysfunction in a mouse model of ASD, related to sensory perception valence (in this case taste). The study is well executed, the data are of high quality, and the analyses procedures are detailed. Furthermore, the behavioural paradigm is well thought, particularly the approach for assessing extinction through repeated retrieval sessions (T1-T5), which effectively tests discrimination between saccharin and water rather than relying solely on lick counts or total consumption as a measure of extinction. Finally, the statistical tests used are appropriate and justified.

Comments on revised version.

The authors have addressed all comments satisfactorily.

# Author comment: Author response

**DOI:** 10.7554/eLife.108988.3.sa4

**Authors:** Chi-Hong Wu, Gina G Turrigiano

**Affiliations:** Brandeis University Waltham United States; Brandeis University Waltham United States

The following is the authors’ response to the original reviews.

**Public Reviews:**

**Reviewer #1 (Public review):**

(1) The experiments rely on three groups: CS-only WT, CTA WT, and CTA KO. Can the authors provide a rationale for not having a CS-only KO group?

We did not include the CS-only (KO) group because longitudinal in vivo recordings in behaving animals are technically demanding, and our primary goal was to follow and compare the dynamics across learning in WT and Shank3 KO mice. However, we recorded an additional habituation water session one day before CST1, which allows us to address (1) the decoding performance in naïve KO animals, and (2) whether higher correlated noise is already present in KO animals before learning. To this end, we first trained and tested the classifier on cross-registered data from the habituation (HAB) water session and the CST1 saccharin session across the CSonly (WT), CTA (WT), and CTA (KO) groups. Because this comparison is made across sessions, it is not exactly comparable to discrimination within a session, but it does show that GC responses in naïve KO animals can discriminate water from saccharin at baseline. These data are now included in Figure 6 – figure supplement 2 and are described in lines 329-338.

Interestingly, we also observed a significant reduction in the amplitude of suppressed responses during the habituation water session in KO compared to WT animals, as well as a trend toward higher stimulus-evoked neuronal coactivity (data included in Figure 2 – figure supplement 2). This suggests that reduced suppression is already present in GC of KO animals before learning, potentially contributing to slower CTA acquisition (now described in lines 209-215).

(2) The authors design an effective behavioral paradigm comparing consumption of water and saccharin and tracking extinction (Figure 3). This paradigm shows differences in licking across distinct behavioral conditions. For instance, during T1, licking to water strongly differs from licking to saccharin for both WT and KO. During T2, licking to water strongly differs from licking to saccharin only for WT (much less for KO), and licking to saccharin in WT differs from that in KO. These differences in taste sampling across conditions could contribute to some of the effects on neural activity and discriminability reported in Figures 5 and 6. That is, sucrose and water trials may be highly discriminable because in one case the mouse licks and in the other it does not (or licks much less). The author may want to address this issue.

This is an important point. As noted by the reviewer, active licking can modulate neuronal activity in the gustatory cortex independent of taste identity (Neese et al., 2022). Because our paradigm required animals to voluntarily sample tastants of different valences, motivated differences in licking are inherently tied to taste value, making it difficult to fully disentangle taste-evoked responses from lick-related activity.

However, taste exposure is known to induce prolonged neural responses that persist beyond the sampling phase (Juen et al., 2024). In our recordings, we included a 10second post-sampling epoch. We thus trained and tested classifiers using calcium traces during this post-sampling period; in particular, we divided the 10-second duration into five 2-second bins, matching the length of the tastant delivery phase, and analyzed the classifier built within each bin. We found that in both WT and KO animals, decoding performance was consistently above chance throughout the postdelivery period (now added to Figure 6 – figure supplement 1, lines 321-329), suggesting that decoding accuracies during sampling likely reflect taste rather than licking.

(3) Are there any omission trials following CTA? If so, they should be quantified and reported. How are the omission trials treated with regard to the analyses?

On the day following each CST session, animals underwent a water-only session (i.e., saccharin was omitted) to minimize context–malaise association. During these sessions, animals resumed licking both in the total lick counts and in the number of trials they engaged in, to levels comparable to pre-conditioning behavior. We did not observe significant differences between the WT and KO groups during these omission sessions. This point has been mentioned in the Methods section of the revised manuscript (lines 544-547).

(4) The authors describe the extinction paradigm as "alternative choice". In decision-making, alternative choice paradigms typically require 2 lateral spouts to report decisions following the sampling from a central spout. To avoid confusion, the authors may want to define their paradigm as alternative sampling.

We have revised this terminology to “alternative sampling” to avoid confusion with the classical alternative-choice paradigms.

(5) Figure 4 reports that CTA increases the proportion of neurons that consistently respond to saccharin and water across days. While the saccharin result could be an effect of aversive learning, it is less clear why the phenomenon would generalize to water as well. Can the authors provide an explanation?

Water and saccharin activated an overlapping population of neurons in GC. When we further quantified their tuning properties in the lifetime plots (Figure 4), we found that neurons responsive to both stimuli showed the most stable responsiveness across days, compared to neurons that responded only to saccharin or only to water (Author response image 1). Because the water-responsive and saccharin-responsive groups in Figure 4 both include this subset of dual-responsive neurons, this likely explains why both plots show increased reliability. This effect on reliability of single-cell responses is thus distinct from changes in the ability to discriminate between tastants at the population level (Fig. 6).

**Author response image 1.** GC neurons responding to both water and saccharin are more stable during CTA extinction. Lifetime plot showing significant responses of the same neurons responding to only water (blue), only saccharin (magenta), and to both saccharin and water (gold) across test sessions (T1-5) in the CTA (WT) group.

(6) The recordings are performed in the part of the anterior insular cortex that is typically defined as "gustatory cortex" (GC). Given the functional heterogeneity of the anterior insular cortex (AIC) and given that the authors do not sample all of the anteroposterior extent of AIC, I would suggest being more explicit about their positioning in GC. Also, some citations (e.g., Gogolla et al, 2014) refer to the posterior insular cortex, which is considered more inherently multimodal than GC. GC multimodality is typically associative in nature, as only a few neurons respond to sound and light in naïve animals.

Our stereotaxic coordinates targeted the conventional gustatory region within AIC (see revised manuscript Methods section, lines 489-490). We have revised the terminology throughout the manuscript to more explicitly reflect this anatomical positioning.

(7) It would be useful to add summary figures showing the extent of viral spread as well as GRIN lens placement.

Revised Manuscript Figure 1B shows a representative example of confirmed GRIN lens placement and the viral spread of GCaMP. In most cases, GCaMP expression is confined to GC, with minimal spread to the piriform cortex and along the injection track. Depth and GCaMP expression in GC were further validated during two-photon imaging.

(8) I encourage the authors to add Ns every time percentages are reported. How many neurons have been recorded in each condition? Can the authors provide the average number of neurons recorded per session and per animal?

We now included these numbers in the revised manuscript (lines 157-158, 162-163, 253, 268, 271-272).

(9) It looks like some animals learned more than others (Figure 1E or Figure 3C). Is it possible to compare neural activity across animals that showed different degrees of learning?

We thank the reviewer for this suggestion – we now show a significant correlation between the magnitude of CTA and the coactivity metric in Figure 1 Figure supplement 3; we elaborate in our Response to Reviewer #3 Public Review 1.

**Reviewer #2 (Public review):**

(1) Causality: The paper infers that increased correlated variability causes learning deficits, but no causal tests (e.g., optogenetic modulation of inhibition or interneuron rescue) are presented to confirm this.

Although we now provide data showing that correlated variability prior to learning is significantly correlated with the magnitude of CTA (see Response to Reviewer #1 Public Review 1above), we agree that we cannot infer causality without additional manipulations. While it might be possible to manipulate correlated variability by targeting inhibition within GC, optogenetic and chemogenetic manipulations of inhibition are likely to impact behavior through multiple mechanisms; for example, enhancing PV-interneuron activity in visual cortex profoundly impairs vision-dependent learning (Bissen et al. 2026, Leman et al. 2025). Thus, testing this would require finding a paradigm that specifically restores synchronization to WT levels without over-inhibiting the network, which is beyond the scope of the current study. We have rewritten the manuscript throughout to remove the inference of causality, and instead describe these two findings as being “associated” (see e.g. lines 95, 219-220, 379-382).

(2) Behavioural scope: The study focuses exclusively on taste aversion; generalisation to other flexible learning paradigms (e.g., reversal or probabilistic tasks) is not addressed.

Our study is focused on conditioned taste aversion (CTA) acquisition and extinction, which provides a well-established model for examining the formation and updating of aversive associative memories. We agree that cognitive flexibility encompasses a broad range of behavioral paradigms, and in the revised manuscript have sought to confined our conclusions to CTA. Whether the mechanisms identified here extend to other forms of flexible learning, such as reversal or probabilistic learning, or even to other sensory-stimulus-guided behavior, will require future investigation.

(3) Mechanistic insights: While providing interesting findings of altered sensory perception and extinction of learning-related signals in AIC, it offered nearly no mechanistic insights. This makes the interpretation, especially on how generalisable these findings are, difficult. Also, different reported findings are "potentially" connected, but the exact relation between increased correlated variability and faster loss of taste selectivity cannot be assessed.

In a new analysis we find that the coactivity level during CST1 showed a significant, positive correlation with the lick ratio (CST2/CST1); that is, the higher the initial coactivity during learning, the slower the CTA acquisition is (Figure 2 – figure supplement 3). This new piece of data (now added to revised manuscript lines 215218) provides a link between these two findings, and suggests that baseline coactivity levels in GC can influence the speed of CTA learning. We agree that this association does not imply causation, and have taken pains to avoid stating this.

**Reviewer #3 (Public review):**

(1) The authors don't make a causal link between the behaviour and AIC neurophysiology, both the percentage of suppressed cells and the coactivity measurements. For the % of suppressed cells, it seems that both WT and KO cells are suppressed in the transition between CST1 and CST2 (Figure 1L), yet only the WT mice exhibit CTA (at least by CST2). For the taste-elicited coactivity measure, it seems that there is an increase in coactivity from CST1 to CST2 in WT (Figure 2C - blue, although not statistically tested?), but persistently higher coactivity in KO. Is this change of coactivity in WT important for the expression of CTA? Plotting behavioral performance (from Figure 1G) against coactivity (from Figure 2C) for each animal would be informative.

This is a good suggestion (also made by the other reviewers), and we now show that the coactivity level during CST1 showed a significant, positive correlation with the lick ratio (CST2/CST1); that is, the higher the initial coactivity during learning, the slower the CTA acquisition is (Figure 2 – figure supplement 3).

(2) Shank3 KO cells already show an increase in baseline coactivity (Figure 2- figure supplement 1), and the authors never examine CS-only responses in the KO group, therefore making it difficult to determine whether elevated coactivity and noise correlations reflect a generalized AIC abnormality in Shank3 KOs (perhaps through impaired PV-mediated inhibition in insular cortex - Gogolla et al, 2014) that is not directly responsible/related to CTA?

We agree that the increased coactivity in KO animals likely reflects a general network defect in AIC before CTA learning. This baseline change is unrelated to the taste stimuli, because (1) the coactivity is already elevated before the animals receive the taste stimuli (that is, a baseline abnormality), (2) the neuronal responses to taste delivery during CST1 is indicative of CS-only responses, as it occurs before the injection of LiCl, when the associative learning process is initiated, and (3) a trend toward higher coactivity is already present in the habituation water session before CST1 (Figure 2 and Figure 2 – figure supplement 2).

We have clarified our description of these findings to avoid claiming that the increased coactivity “causes” poor learning performance (lines 379-382).

(3) How do the authors interpret the large range of lick ratios (Figure 1G) for WT (almost bi-modal distribution)? Is there a within-subject correlation with any of the neurophysiological measurements to suggest a relationship between AIC neurophysiology and behavioural expression of CTA?

See response to Point 1 above.

(4) Indeed, CTA appears to be successfully achieved for Shank3 KO mice delayed by 1 day, as the level of saccharin aversion during the first retrieval session (T1) is comparable between Shank3 KO and WTs. In this context, not extending the first part of the paradigm to include CST3 seems to be a missed opportunity. Doing so would have allowed for within-cell and within-subject comparison of taste-elicited pairwise correlation across the learning and to investigate the neural mechanism of delayed extinction in KOs more effectively.

We did not include a third CST session because when we analyzed the lick counts, KO animals already formed robust CTA after CST2 that was indistinguishable from WT animals. This suggests that the faster loss of CTA memory during extinction is due to a faster extinction process, rather than a weaker CTA memory from the outset. Adding a third CST could potentially lead to a memory that is harder to extinguish. Whether Shank3 KO mice would exhibit faster loss of memory in this scenario is an open question that would be interesting to explore in a future study.

(5) How to interpret Figure 5F: Absolute discriminability is lower for T5 for CTA WT and CTA KO compared to CS-only? Why would AIC neurons have less information on taste identity by the end of extinction than during the unconditioned (CS-only) condition? And if that is the case, how is decoding accuracy in Figure 6C higher in T5 for CTA WT vs CS-only?

We appreciate the reviewer's confusion about the discrepancy between our single-cell and population-level discriminability results. We speculate that in the CS-only state, individual AIC neuronal responses mostly reflect taste identity. However, after learning (in the CTA group), these neurons develop “mixed selectivity” (Tye et al., 2024), encoding not only identity but also the learned valence and extinction history. The lower single-cell discriminability after extinction (T5) in Figure 5F suggests that, although taste identity may remain constant, the learned history (e.g., "this taste used to be dangerous, but now it's safe") has shifted. This mixing of information makes each cell a weaker discriminator on its own.

However, the higher population decoding accuracy in Figure 6C demonstrates that the entire population of neurons can work together more effectively. The learning process could reorganize the neural ensemble in such ways that our support vector classifier (SVC) is able to identify and combine the relevant signals within the population, even when the valence of taste stimuli has changed, to better decode stimuli and outperform the non-learned state. This suggests that the brain shifted to a more robust, population-based coding strategy for complex, learned information, which is resistant to changes in selectivity at the single-cell level. The finding that population coding is robust to single-neuron variability has also been reported in other cortical regions (Montijin et al., 2016).

**Recommendations for the authors:**

**Reviewer #2 (Recommendations for the authors):**

(1) Mechanistic experiments: Consider inhibitory neuron-specific imaging or manipulation (e.g., optogenetic enhancement of interneuron activity) to test whether restoring inhibition rescues learning flexibility.

We have addressed the limitation and potential issues for manipulating cortical inhibition in Response to Reviewer #2 Public review 1.

(2) Clarify limitations: Explicitly acknowledge the correlational nature of neuralbehavioural relationships in the Discussion.

We have removed language that implies a causal relationship throughout, and have emphasized the correlational nature of our findings in the Discussion section of our revised manuscript (lines 379-382)

(3) Enhance clarity: Simplify some dense methodological sections and expand figure legends to guide interdisciplinary readers.

We have adjusted the Methods section and figure legends as needed for better readability.

Individual Comments for Authors:

(1) L83-90: Confusingly written, not easy to understand for someone not knowing the paradigm in detail.

- What are the different stages? Memory encoding? Leaning? Extinction

- More reliable in taste responsiveness - what does that mean?

We have emphasized the behavior stages where each finding was observed in the revised manuscript (lines 82-91)

(2) L112: Not sure if these references support the "crucial", since they do not seem to be causal.

We have reworded this for accuracy (line 111).

(3) Figure 1: panels h and i in the heat maps, it looks like that in the KO animal, activity is more suppressed from CST1 to 2?

Panels j, l, m, and Figure 2: Neuronal suppression is already higher in CST1; therefore, there is no CTA effect but a general "perceptual" issue in the Shank3 model. The only effect seems to be a potential reduction in activation in CST2 in KO animals.

This point has been discussed in Reviewer #3 Public Review 2.

(4) Clarify in text. Especially with the sentence in the next paragraph, it might be confusing: "We wondered what other features of AIC activity during CTA acquisition might differ between WT and Shank3 KO mice."

We have rewritten this in the revised manuscript (lines 169-170).

(5) Clarify which are CTA-dependent and which are general e.g., if writing suppression during CTA acquisition, it implies that it is related. But these changes were present before CTA.

We have clarified this in the revised manuscript (lines 209-215).

(6) Figure 4: Mainly shows a CTA-related increase in reliability in their taste responsiveness. This is not addressed anywhere else in the document and is not taken up in the discussion. How could it be related to the other findings, and what is its relevance? Please elaborate (e.g., in the discussion) or potentially remove?

We measured response reliability, as stabilization of stimulus-evoked responses has been reported in other sensory cortices across different learning tasks. Yet, it remained unclear whether CTA learning would induce similar changes in AIC. We took advantage of our longitudinal recording to address this question and believe that this piece of evidence will contribute to the research community that studies taste and learning in general. In addition, what is striking to us is that while the taste selectivity is degraded faster in KO animals, their response reliability is largely preserved. This suggests that these two sensory stimulus-related neuronal properties may involve distinct cellular and/or circuit mechanisms.

(7) Figure 5: Problematic to compare T5 between both groups, since T5 is lower than T4 in WT (against the trend) and T4 is an outlier in KO. e.g., if compared at T5, completely different results? Or why is there significance between T1 and T2 but not between T1 and T4 in KO? Could the authors address this point?

In Figure 5B, the slightly lower average for WT animals at T5 was driven by a single outlier, and there was no statistically significant difference between T4 and T5 (corrected post hoc t-test, WT, T4 vs. T5, p = 0.4097). Therefore, it does not contradict the trend toward an overall increase in nonselective neurons during CTA extinction. For KO animals, the lower average at T4 than T5 (corrected post hoc ttest, KO, T4 vs. T5, p = 0.0082) was intriguing, and one possible explanation is that neurons in the KO group might undergo more dynamic and variable changes in their responsiveness during CTA extinction, fluctuating before finally stabilizing.

Comparing T5 instead of T4 thus ensures that neuronal responsiveness is stabilized and reflects an “extinct” CTA memory more truly.

General Comments:

(1) While changes in SNR were observed in Shank3 models, the mechanism underlying decreased correlated variability has not been reported to date. Since decreased variability is usually associated with improved SNR ratio, it might be worth highlighting the distinction between "signal" and "noise" as separated in your analyses to make it more understandable for the reader.

We have described in the Results section what signal and noise correlations indicated and how they were separated in our analyses in both the Results and Methods section of the revised manuscript (lines 185-193, lines 673-681).

(2) What is the origin of the increased correlated variability?

We have discussed that reduced cortical feedback inhibition could be a potential source of increased correlated variability in the Discussion section of our revised manuscript (lines 370-375).

(3) Is the variability generally increased between trials (bigger fluctuations between trials for each neuron), or is the variability of each neuron similar, but they are just more correlated (more synced)?

Our pilot analysis did not detect any evident changes in the response variability for each neuron across trials; thus, we think that in KO animals, neuronal responsivity becomes more correlated and synchronized.

**Reviewer #3 (Recommendations for the authors):**

(1) Point in line 422-424: Rephrase the closing statement of the discussion as you have shown that mutant mice are actually able to update their behaviour (in fact faster) when the valence of the sensory input changes.

The “reduced ability to update behavior when the valence of a sensory input changes” refers to the finding that KO animals learned CTA more slowly; i.e., they were unable to timely adjust their behavior after malaise. We have rephrased this for clarity (line 448-449)

(2) The Figure 6 legend does not correspond to panels D and E in the figure. Νο I, J in figure.

We have fixed this mismatch in the revised manuscript.

Minor concerns:

(1) Cue/lick/taste-responding neurons greatly overlap and are not exclusively selective (Figure 1- figure supplement 2). Is there a genotype difference for the % of selective neurons (i.e., ones that only respond during cut/lick/taste) or the % of overlap?

When we quantified the stimulus responsivity in KO animals, we also identified neurons that were activated by cues, licks, or tastes. Their respective percentages and overlap did not differ significantly from those in the WT group, indicating that the modality of KO neurons across different sensorimotor cues is not compromised in the KO condition (Author response image 2).

**Author response image 2.** Neurons in WT and Shank3 KO animals show comparable responsiveness to sensorimotor stimuli during conditioning. (A) Percentage of neurons activated by the cue (left), lick movement (middle), and the tastant (right) in the CTA (KO) group (B) during the first conditioning session (CST1). (B) Venn diagram showing the overlaps among cue-, lick-, and tastant responsive neurons in (Figure 1 - figure supplement 2 C) and (A).

(2) For Figure 1: The authors could also express consumption as a % of consumed (trial-averaged licks) over the number of trials. It is mentioned that mice undergo daily training sessions consisting of 'approximately 30 trials' (line 114). This can give an indication of how strong the learning is between cta1 and cta2 and how strong the genotype difference is.

We are not sure if dividing trial-averaged licks over the number of trials would provide additional information, as the trial-average lick is already normalized to the number of trials.

(3) Figure 4: Why is there a different number of neurons in C vs G?

The figures B, C, D showed neurons that were activated by saccharin, and the figures F, G, H showed neurons that were activated by water. In all experimental groups, the numbers of neurons responsive to saccharin and water were different (i.e., B vs. F, C vs. G, D vs. H). The exact numbers were included in the corresponding figure legends in the revised manuscript.

(4) Figure 5B: The grey background box is moved to the left.

We kept the current figure format, as it effectively presents the mean, fitted mean, error bars, and individual animal data.

(5) In line 142: (1-2), (2-3), (3-4), the numbers in parentheses are confusing.

We have relabeled this as epoch 1-2, epoch 2-3, and epoch 3-4 in both text and figures for clarity (lines 145-146).

(6) Line 188: Do the authors mean noise correlations?

Rosenbaum et al. and Khoury et al. indeed measured correlated variability (noise correlation) in their study. On the other hand, Rothschild et al. did not specifically separate the noise from signal activities, which more likely reflect the coactivity measured in our case. We have rewritten this for accuracy (line 196).

(7) Where mentioning in the CS-only group, please explicitly state the CS-only WT group.

We have relabeled this throughout our revised manuscript.

(8) In lines 273-274: if the comparison is the reduction in discriminability being faster for the KO animals that had CTA, the correct comparison should be CSonly KO vs CTA KO.

We think that the better comparison to test how fast taste discriminability is reduced would be to perform post-hoc tests comparing T1 vs T2 within genotypes. We did not see significant changes between T1 and T2 in either genotype, which was reported in the figure legends of the reviewed preprint (lines 1140-1141).
